# Supplementary material for: Physician-Led Thoracic Trauma Management in a Specialist Emergency Care Centre
Source: J Clin Med. 2021 Dec 11;10(24):5806. doi: 10.3390/jcm10245806 (PMC8709173; doi:10.3390/jcm10245806)
Supplement: Supplementary file 1 [file jcm-10-05806-s001.zip › jcm-1451786-supplementary.pdf]

Table S1: comparison of variables between those under 65 years of age to those over 65 years.

	Age under 65 years	Age above 65 years of age	*P* value
**Number of patients**	36	83	not applicable
**Number with no comorbidities**	12	7	0.0084
**Number with 1 comorbidity**	12	3	0.0003
**Number with more than 1 comorbidity**	12	73	0.0076
**Trauma assessment (Pan CT or full clinical assessment)**	23	52	1
**Falls from standing (less than 2m)**	5	79	0.00001
**Average number of rib fractures**	4.25 (0-9)	3.4 (0-10)	0.499
**Length of stay (days)**	Mean 5, median 4 (Range 1–14, IQR 5.5)	Mean 8.4, median 6 (Range 1–54, IQR 7)	
**Incidence of pneumonia (total number)**	4	21	0.22
**Mortality (total number)**	0	16	0.006
